# Diet in Typhoid Fever

**Published:** 1900-02

**Authors:** 


### Abstracts.

### DIET IN TYPHOID FEVER.

Shurtleff, Fred C., (*Southern California Practitioner*,) Los Angeles, Cal., says: Much has been written both pro and con in reference to this or that article for diet in the management of typhoid fever. It is a settled fact that the food must be fluid, highly nutritious and easy of digestion, for the maintenance of nutrition is imperative in this wasting disease. Milk is probably the most extensively used and will form the main article of diet so long as fever lasts. I have used milk in nearly all its various forms in the care of my cases from frozen or boiled sweet milk to butter milk, from sweet milk, or milk with lime water, to that partially digested with pepsin or pancreatin when digestion was enfeebled. The tendency in milk diet is to overfeed by forcing too large quantities at one feeding and thereby cause disgust for that diet upon which we have pinned our faith. If one insists upon an absolute milk diet, not infrequently will you find your patient has gone without it rather than take it. Patients fret under its administration, digestion is interfered with, curds swarming with bacteria of decomposition are found in the increased diarrheal discharges, plus the bacteria of typhoid fever already existing, hence the object which we wish to attain so far as it is possible—that of rendering the gastro-intestinal tract aseptic—is defeated from the outset by error in diet. I have often been puzzled as to what to substitute for milk in this class of cases until the stomach became more tolerant. I have tried various farinaceous substances and discarded them on account of the increase of flatulency they almost invariably produced.

For some time past I have tided my patients over their critical period by tablespoonful doses of liquid peptonoids every two hours, giving nothing else in the way of nourishment but the above remedy. I cannot speak too highly of this elegant preparation where digestion is below par, as a highly nutritious food that will not curdle upon the stomach or leave a residue in the intestinal tract. It is a slightly stimulating food, consequently patients, as a rule, will require less alcoholic stimulants, a great desideratum in some cases. I do frequently carry through my cases of typhoid successfully, where no other article of diet is given from the time I make the diagnosis until convalescence is firmly established, and I call the attention of the profession to it for that class of cases in which milk cannot be taken.

